# *trans*-Tetra­carbonyl­bis­(tri­phenyl­phosphane-κ*P*)molybdenum(0)

**DOI:** 10.1107/S1600536814000300

**Published:** 2014-01-11

**Authors:** Greyson W. Waldhart, Neal P. Mankad

**Affiliations:** a Department of Chemistry, University of Illinois at Chicago, 845 West Taylor Street, Chicago, IL 60607, USA

## Abstract

The well known title compound, *trans*-[Mo(C_18_H_15_P)_2_(CO)_4_], has not been studied previously by X-ray crystallography, unlike its *cis* isomer. The complex possesses crystallographically imposed inversion symmetry, with the Mo atom residing on an inversion centre (1*a* Wyckoff position). The two tri­phenyl­phosphane groups are arranged in a staggered orientation. Each of the phenyl groups exhibits significantly different Mo—P—C—C torsion angles ranging from 2.6 (2) to 179.4 (1)°, most likely due to steric inter­actions based upon their positions relative to the carbonyl ligands.

## Related literature   

For the synthesis of the title compound and a structural study of its *cis* isomer, see: Cotton *et al.* (1982 ▶). For ligand dissociation and thermal reactivity of similar compounds, see: Darensbourg & Kump (1978 ▶). For an IR analysis of metal carbonyls, see: Haas & Sheline (1967 ▶). For kinetic investigations of metal–phosphanes, see: Darensbourg & Bischoff (1993 ▶).
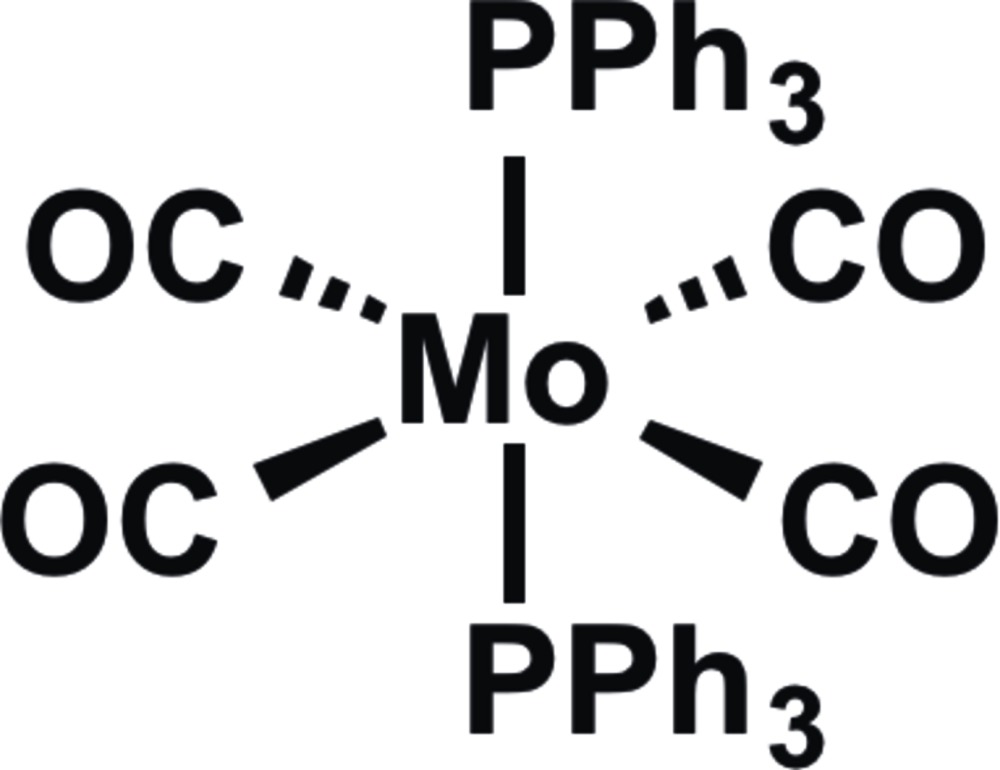


## Experimental   

### 

#### Crystal data  

[Mo(C_18_H_15_P)_2_(CO)_4_]
*M* *_r_* = 732.52
Triclinic, 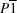

*a* = 9.3443 (13) Å
*b* = 10.2267 (15) Å
*c* = 10.7258 (16) Å
α = 64.794 (4)°
β = 69.417 (4)°
γ = 83.699 (4)°
*V* = 867.2 (2) Å^3^
*Z* = 1
Mo *K*α radiation
μ = 0.51 mm^−1^
*T* = 300 K
0.62 × 0.45 × 0.33 mm

#### Data collection  

Bruker SMART X2S benchtop diffractometer
Absorption correction: multi-scan (*SADABS*; Bruker, 2012 ▶) *T*_min_ = 0.70, *T*_max_ = 0.85
7865 measured reflections
2907 independent reflections
2770 reflections with *I* > 2σ(*I*)
*R*_int_ = 0.020

#### Refinement  

*R*[*F* ^2^ > 2σ(*F* ^2^)] = 0.022
*wR*(*F* ^2^) = 0.058
*S* = 1.11
2907 reflections
214 parameters
61 restraints
H-atom parameters constrained
Δρ_max_ = 0.25 e Å^−3^
Δρ_min_ = −0.28 e Å^−3^

### 

Data collection: *SMART* (Bruker, 2007 ▶); cell refinement: *SAINT* (Bruker, 2013 ▶); data reduction: *SAINT*; program(s) used to solve structure: *SHELXS97* (Sheldrick, 2008 ▶); program(s) used to refine structure: *SHELXL2013* (Sheldrick, 2008 ▶); molecular graphics: *Mercury* (Macrae *et al.*, 2006 ▶); software used to prepare material for publication: *publCIF* (Westrip, 2010 ▶).

## Supplementary Material

Crystal structure: contains datablock(s) global, I. DOI: 10.1107/S1600536814000300/pj2007sup1.cif

Structure factors: contains datablock(s) I. DOI: 10.1107/S1600536814000300/pj2007Isup2.hkl

Supporting information file. DOI: 10.1107/S1600536814000300/pj2007Isup3.cml

CCDC reference:

Additional supporting information:  crystallographic information; 3D view; checkCIF report

## Figures and Tables

**Fig. 1.**
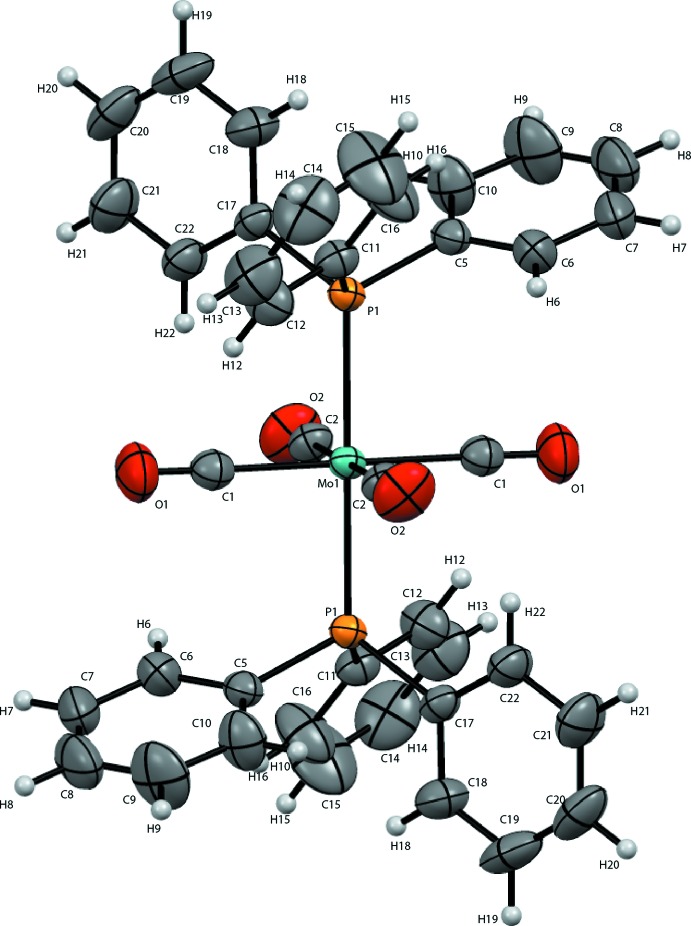
Thermal ellipsoid plot of the title compound (50% probability).

## supplementary crystallographic information

### 1. Comment

We initiated this study as part of an undergraduate teaching lab using the Bruker *SMART* X2S bench-top diffractometer. Students use FT—IR spectroscopy to propose whether unknown samples are either the *cis*- or *trans*-isomer, and then use crystallography to test their hypotheses. To our surprise, the *trans*-isomer had not been reported in the CSD. In our hands, dissolving the *trans*-isomer in dichloromethane causes reversion to the *cis*-isomer. Crystallization from chloroform, on the other hand, provides the *trans* arrangement cleanly.

The 0 1 0 and 0 0 1 reflections were omitted from final refinements because of the suspicion that they were affected by the beamstop. Hydrogen atoms were placed at calculated positions 0.93 angstroms from the phenyl carbons and refined using the standard riding model with U_iso_(H) set to 1.2 times U_eq_(C).

### Crystal data

**Table:**

[Mo(C_18_H_15_P)_2_(CO)_4_]	*Z* = 1
*M**_r_* = 732.52	*F*(000) = 374
Triclinic, *P*1	*D*_x_ = 1.403 Mg m^−^^3^
*a* = 9.3443 (13) Å	Mo *K*α radiation, λ = 0.71073 Å
*b* = 10.2267 (15) Å	Cell parameters from 6345 reflections
*c* = 10.7258 (16) Å	θ = 2.2–25.1°
α = 64.794 (4)°	µ = 0.51 mm^−^^1^
β = 69.417 (4)°	*T* = 300 K
γ = 83.699 (4)°	Block, yellow
*V* = 867.2 (2) Å^3^	0.62 × 0.45 × 0.33 mm

### Data collection

**Table:**

Bruker SMART X2S benchtop diffractometer	2907 independent reflections
Radiation source: XOS X-beam microfocus source	2770 reflections with *I* > 2σ(*I*)
Doubly curved silicon crystal monochromator	*R*_int_ = 0.020
Detector resolution: 8.3330 pixels mm^-1^	θ_max_ = 24.7°, θ_min_ = 2.3°
ω scans	*h* = −10→10
Absorption correction: multi-scan (*SADABS*; Bruker, 2012)	*k* = −12→12
*T*_min_ = 0.70, *T*_max_ = 0.85	*l* = −12→12
7865 measured reflections	

### Refinement

**Table:**

Refinement on *F*^2^	Primary atom site location: structure-invariant direct methods
Least-squares matrix: full	Secondary atom site location: difference Fourier map
*R*[*F*^2^ > 2σ(*F*^2^)] = 0.022	Hydrogen site location: inferred from neighbouring sites
*wR*(*F*^2^) = 0.058	H-atom parameters constrained
*S* = 1.11	*w* = 1/[σ^2^(*F*_o_^2^) + (0.0238*P*)^2^ + 0.3878*P*] where *P* = (*F*_o_^2^ + 2*F*_c_^2^)/3
2907 reflections	(Δ/σ)_max_ = 0.001
214 parameters	Δρ_max_ = 0.25 e Å^−^^3^
61 restraints	Δρ_min_ = −0.28 e Å^−^^3^

### Special details

**Table:**

Experimental. For synthesis of the compound, see Cotton (1982). Yellow crystals of the title compound suitable for X-ray diffraction were obtained by layering methanol above a chloroform solution of the title compound and allowing the layers to mix gradually. This crystallization method was performed on the bench with reagent grade solvents and without use of an inert atmosphere.
Geometry. All e.s.d.'s (except the e.s.d. in the dihedral angle between two l.s. planes) are estimated using the full covariance matrix. The cell e.s.d.'s are taken into account individually in the estimation of e.s.d.'s in distances, angles and torsion angles; correlations between e.s.d.'s in cell parameters are only used when they are defined by crystal symmetry. An approximate (isotropic) treatment of cell e.s.d.'s is used for estimating e.s.d.'s involving l.s. planes.
Refinement. Refinement of *F*^2^ against ALL reflections. The weighted *R*-factor *wR* and goodness of fit *S* are based on *F*^2^, conventional *R*-factors *R* are based on *F*, with *F* set to zero for negative *F*^2^. The threshold expression of *F*^2^ > σ(*F*^2^) is used only for calculating *R*-factors(gt) *etc*. and is not relevant to the choice of reflections for refinement. *R*-factors based on *F*^2^ are statistically about twice as large as those based on *F*, and *R*- factors based on ALL data will be even larger.

### Fractional atomic coordinates and isotropic or equivalent isotropic displacement parameters (Å^2^)

**Table:**

	*x*	*y*	*z*	*U*_iso_*/*U*_eq_	
Mo1	0	0	1.0	0.02814 (8)	
P1	0.21394 (5)	0.16894 (5)	0.79865 (5)	0.02888 (12)	
O1	0.0078 (2)	0.1374 (2)	1.21320 (19)	0.0659 (5)	
O2	0.2517 (2)	−0.22032 (19)	1.0879 (2)	0.0677 (5)	
C1	0.0065 (2)	0.0886 (2)	1.1355 (2)	0.0396 (4)	
C2	0.1594 (2)	−0.1425 (2)	1.0585 (2)	0.0400 (4)	
C5	0.2591 (2)	0.3109 (2)	0.8425 (2)	0.0360 (4)	
C6	0.3274 (3)	0.2769 (3)	0.9478 (2)	0.0516 (5)	
H6	0.3582	0.1834	0.99	0.062*	
C7	0.3502 (3)	0.3800 (3)	0.9906 (3)	0.0643 (7)	
H7	0.3976	0.3557	1.0602	0.077*	
C8	0.3043 (4)	0.5164 (3)	0.9327 (3)	0.0750 (8)	
H8	0.3201	0.5855	0.9619	0.09*	
C9	0.2344 (4)	0.5514 (3)	0.8306 (4)	0.0842 (9)	
H9	0.2014	0.6445	0.7915	0.101*	
C10	0.2122 (3)	0.4496 (3)	0.7848 (3)	0.0604 (6)	
H10	0.1654	0.4753	0.7146	0.072*	
C11	0.3989 (2)	0.0886 (2)	0.7434 (2)	0.0375 (4)	
C12	0.3988 (3)	−0.0338 (2)	0.7195 (3)	0.0527 (6)	
H12	0.3059	−0.0751	0.7371	0.063*	
C13	0.5324 (3)	−0.0962 (3)	0.6703 (3)	0.0684 (7)	
H13	0.5289	−0.1772	0.6529	0.082*	
C14	0.6677 (3)	−0.0407 (4)	0.6475 (3)	0.0800 (9)	
H14	0.7578	−0.0844	0.6167	0.096*	
C15	0.6720 (3)	0.0795 (4)	0.6694 (4)	0.0992 (12)	
H15	0.7659	0.119	0.6516	0.119*	
C16	0.5381 (3)	0.1452 (3)	0.7183 (4)	0.0735 (8)	
H16	0.5431	0.2271	0.7339	0.088*	
C17	0.1953 (2)	0.27353 (19)	0.61689 (19)	0.0348 (4)	
C18	0.3120 (3)	0.3699 (2)	0.5024 (2)	0.0542 (6)	
H18	0.4008	0.3822	0.5174	0.065*	
C19	0.2971 (3)	0.4474 (3)	0.3666 (3)	0.0668 (7)	
H19	0.3751	0.5128	0.2912	0.08*	
C20	0.1676 (4)	0.4284 (3)	0.3421 (2)	0.0657 (7)	
H20	0.1576	0.4813	0.2506	0.079*	
C21	0.0550 (3)	0.3322 (3)	0.4515 (3)	0.0659 (7)	
H21	−0.0316	0.3174	0.4346	0.079*	
C22	0.0681 (3)	0.2555 (2)	0.5893 (2)	0.0485 (5)	
H22	−0.0109	0.1909	0.6641	0.058*	

### Atomic displacement parameters (Å^2^)

**Table:**

	*U*^11^	*U*^22^	*U*^33^	*U*^12^	*U*^13^	*U*^23^
Mo1	0.02853 (13)	0.02430 (12)	0.02774 (12)	−0.00060 (8)	−0.00954 (9)	−0.00693 (9)
P1	0.0288 (3)	0.0261 (2)	0.0290 (2)	−0.00119 (18)	−0.00896 (19)	−0.00910 (19)
O1	0.0699 (12)	0.0822 (12)	0.0664 (11)	0.0004 (9)	−0.0223 (9)	−0.0497 (10)
O2	0.0675 (11)	0.0606 (10)	0.0694 (11)	0.0316 (9)	−0.0346 (10)	−0.0197 (9)
C1	0.0355 (11)	0.0402 (11)	0.0406 (11)	−0.0005 (8)	−0.0115 (9)	−0.0150 (9)
C2	0.0432 (12)	0.0351 (10)	0.0358 (10)	0.0027 (9)	−0.0130 (9)	−0.0100 (8)
C5	0.0347 (10)	0.0355 (10)	0.0353 (10)	−0.0054 (8)	−0.0057 (8)	−0.0156 (8)
C6	0.0599 (14)	0.0485 (12)	0.0477 (12)	−0.0108 (10)	−0.0196 (11)	−0.0170 (10)
C7	0.0701 (17)	0.0782 (18)	0.0525 (14)	−0.0245 (14)	−0.0167 (12)	−0.0311 (13)
C8	0.088 (2)	0.0738 (19)	0.0778 (19)	−0.0188 (15)	−0.0106 (16)	−0.0531 (16)
C9	0.110 (3)	0.0520 (16)	0.115 (3)	0.0194 (16)	−0.046 (2)	−0.0537 (17)
C10	0.0742 (17)	0.0479 (13)	0.0787 (17)	0.0172 (12)	−0.0379 (14)	−0.0379 (13)
C11	0.0330 (10)	0.0387 (10)	0.0354 (10)	0.0038 (8)	−0.0102 (8)	−0.0122 (8)
C12	0.0471 (13)	0.0524 (13)	0.0599 (14)	0.0043 (10)	−0.0101 (11)	−0.0314 (11)
C13	0.0693 (18)	0.0652 (16)	0.0713 (17)	0.0220 (13)	−0.0159 (14)	−0.0399 (14)
C14	0.0568 (17)	0.099 (2)	0.087 (2)	0.0381 (16)	−0.0221 (15)	−0.0508 (19)
C15	0.0332 (14)	0.136 (3)	0.151 (3)	0.0142 (17)	−0.0281 (18)	−0.085 (3)
C16	0.0379 (13)	0.0846 (19)	0.114 (2)	0.0031 (12)	−0.0185 (14)	−0.0620 (19)
C17	0.0421 (11)	0.0287 (9)	0.0281 (9)	0.0027 (8)	−0.0091 (8)	−0.0094 (7)
C18	0.0534 (14)	0.0503 (13)	0.0410 (12)	−0.0077 (10)	−0.0067 (10)	−0.0080 (10)
C19	0.0797 (19)	0.0506 (14)	0.0345 (12)	−0.0025 (13)	0.0013 (12)	−0.0006 (10)
C20	0.092 (2)	0.0577 (15)	0.0327 (12)	0.0155 (14)	−0.0212 (13)	−0.0079 (11)
C21	0.0777 (18)	0.0697 (16)	0.0470 (13)	0.0055 (14)	−0.0362 (13)	−0.0097 (12)
C22	0.0520 (13)	0.0496 (12)	0.0361 (11)	−0.0042 (10)	−0.0186 (10)	−0.0062 (9)

### Geometric parameters (Å, º)

**Table:**

Mo1—C1	2.034 (2)	C11—C16	1.373 (3)
Mo1—C1^i^	2.034 (2)	C11—C12	1.380 (3)
Mo1—C2^i^	2.035 (2)	C12—C13	1.376 (3)
Mo1—C2	2.035 (2)	C12—H12	0.93
Mo1—P1^i^	2.4879 (5)	C13—C14	1.343 (4)
Mo1—P1	2.4879 (5)	C13—H13	0.93
P1—C5	1.8341 (19)	C14—C15	1.353 (4)
P1—C11	1.8430 (19)	C14—H14	0.93
P1—C17	1.8430 (19)	C15—C16	1.395 (4)
O1—C1	1.143 (3)	C15—H15	0.93
O2—C2	1.141 (2)	C16—H16	0.93
C5—C10	1.376 (3)	C17—C22	1.370 (3)
C5—C6	1.389 (3)	C17—C18	1.390 (3)
C6—C7	1.378 (3)	C18—C19	1.381 (3)
C6—H6	0.93	C18—H18	0.93
C7—C8	1.354 (4)	C19—C20	1.374 (4)
C7—H7	0.93	C19—H19	0.93
C8—C9	1.367 (4)	C20—C21	1.352 (4)
C8—H8	0.93	C20—H20	0.93
C9—C10	1.386 (3)	C21—C22	1.390 (3)
C9—H9	0.93	C21—H21	0.93
C10—H10	0.93	C22—H22	0.93
			
C1—Mo1—C1^i^	180.00 (11)	C5—C10—H10	119.8
C1—Mo1—C2^i^	89.09 (8)	C9—C10—H10	119.8
C1^i^—Mo1—C2^i^	90.91 (8)	C16—C11—C12	117.4 (2)
C1—Mo1—C2	90.91 (8)	C16—C11—P1	124.63 (17)
C1^i^—Mo1—C2	89.09 (8)	C12—C11—P1	117.92 (16)
C2^i^—Mo1—C2	180.0	C13—C12—C11	121.8 (2)
C1—Mo1—P1^i^	89.67 (6)	C13—C12—H12	119.1
C1^i^—Mo1—P1^i^	90.33 (6)	C11—C12—H12	119.1
C2^i^—Mo1—P1^i^	87.97 (6)	C14—C13—C12	120.3 (3)
C2—Mo1—P1^i^	92.03 (6)	C14—C13—H13	119.9
C1—Mo1—P1	90.33 (6)	C12—C13—H13	119.9
C1^i^—Mo1—P1	89.67 (6)	C13—C14—C15	119.4 (3)
C2^i^—Mo1—P1	92.04 (6)	C13—C14—H14	120.3
C2—Mo1—P1	87.97 (6)	C15—C14—H14	120.3
P1^i^—Mo1—P1	180.0	C14—C15—C16	121.2 (3)
C5—P1—C11	104.37 (9)	C14—C15—H15	119.4
C5—P1—C17	102.38 (9)	C16—C15—H15	119.4
C11—P1—C17	99.19 (9)	C11—C16—C15	119.8 (3)
C5—P1—Mo1	112.63 (6)	C11—C16—H16	120.1
C11—P1—Mo1	116.61 (6)	C15—C16—H16	120.1
C17—P1—Mo1	119.47 (6)	C22—C17—C18	117.96 (19)
O1—C1—Mo1	178.84 (18)	C22—C17—P1	121.05 (14)
O2—C2—Mo1	178.21 (19)	C18—C17—P1	120.96 (16)
C10—C5—C6	117.94 (19)	C19—C18—C17	120.5 (2)
C10—C5—P1	121.47 (16)	C19—C18—H18	119.8
C6—C5—P1	120.24 (16)	C17—C18—H18	119.8
C7—C6—C5	120.8 (2)	C20—C19—C18	120.4 (2)
C7—C6—H6	119.6	C20—C19—H19	119.8
C5—C6—H6	119.6	C18—C19—H19	119.8
C8—C7—C6	120.8 (3)	C21—C20—C19	119.7 (2)
C8—C7—H7	119.6	C21—C20—H20	120.2
C6—C7—H7	119.6	C19—C20—H20	120.2
C7—C8—C9	119.3 (2)	C20—C21—C22	120.3 (3)
C7—C8—H8	120.3	C20—C21—H21	119.9
C9—C8—H8	120.3	C22—C21—H21	119.9
C8—C9—C10	120.7 (3)	C17—C22—C21	121.2 (2)
C8—C9—H9	119.6	C17—C22—H22	119.4
C10—C9—H9	119.6	C21—C22—H22	119.4
C5—C10—C9	120.4 (2)		

Symmetry code: (i) −*x*, −*y*, −*z*+2.
